# General and sport-specific mental toughness in university students: associations with personality traits and physical activity

**DOI:** 10.3389/fpsyg.2026.1854907

**Published:** 2026-06-16

**Authors:** Debora Di Mauro, Carmen Mannucci, Alessandro Coronella, Rosa Angela Fabio

**Affiliations:** Department of Biomedical, Dental and Morphological and Functional Imaging Sciences, University of Messina, Messina, Italy

**Keywords:** competitive anxiety, mental toughness, personality traits, physical activity, university students

## Abstract

**Objectives:**

This study aimed to examine general and sport-specific mental toughness in university students and their associations with personality traits, sport participation, and academic performance, as well as competitive anxiety in students engaged in sport.

**Methods:**

This cross-sectional study involved 239 university students. Participants completed the Mental Toughness Questionnaire-18 (MTQ-18), the Sport Mental Toughness Questionnaire (SMTQ), and the Big Five Inventory (BFI). Demographic, academic, and behavioral variables were also collected, and students engaged in regular sport practice additionally completed the Sport Competition Anxiety Test (SCAT).

**Results:**

General and sport-specific mental toughness were strongly positively associated, indicating substantial overlap between the two constructs while preserving domain-specific features. Conscientiousness emerged as the main positive predictor of both forms of mental toughness. Academic performance was positively associated with general mental toughness. Training frequency was positively associated with both general and sport-specific mental toughness. Among students engaged in sport practice, higher sport-specific mental toughness was associated with lower competitive anxiety, while neuroticism was positively related to anxiety levels. Daily phone use and sedentary behavior were examined as additional behavioral correlates.

**Conclusions:**

Mental toughness in university students appears to be associated primarily with dispositional factors, particularly conscientiousness, and with sport participation variables such as training frequency. General mental toughness was also positively linked to academic functioning, whereas competitive anxiety emerged as a relevant correlate among students engaged in regular sport practice.

## 1 Introduction

Mental toughness is a central construct in sport psychology and is commonly described as a psychological resource that supports persistence, confidence, emotional control, and effective functioning under pressure (Jones et al., 2002; Clough et al., 2002). Although originally developed within sport settings, the construct has progressively been extended to broader domains of functioning, including self-regulation, coping, and goal pursuit in everyday life (Clough and Strycharczyk, 2012; Gucciardi, 2014). Current perspectives converge in treating mental toughness as a multidimensional construct rather than a single trait, with particular emphasis on commitment, challenge, control, and confidence (Clough et al., 2002; Gucciardi et al., 2008, 2009). Recent systematic reviews and meta-analytic studies have further confirmed the relevance of mental toughness in sport psychology, particularly in relation to performance, coping, and psychological skills development (Stamatis et al., 2020).

Research has shown that mental toughness is associated with adaptive functioning in demanding contexts, including performance maintenance, emotional regulation, and cognitive control (Dewhurst et al., 2019; Welsh et al., 2023). However, the extent to which mental toughness reflects a broad dispositional tendency or a context-specific resource remains debated. This issue is especially relevant when comparing everyday functioning with sport participation, where evaluative pressure, competition, and repeated exposure to challenge may shape the expression of the construct in specific ways (Gucciardi et al., 2008; Sheard et al., 2009).

A useful distinction in this literature is that between general and sport-specific mental toughness. General mental toughness refers to a broader tendency to cope effectively with demands across life domains, whereas sport-specific mental toughness refers more directly to psychological functioning in athletic settings characterized by pressure, competition, and performance evaluation (Sheard et al., 2009). Examining both dimensions within the same study may clarify whether sport-specific mental toughness largely reflects a domain-specific manifestation of a broader psychological disposition or whether it captures more distinct processes linked to sport participation (Clough et al., 2002; Gucciardi et al., 2008; Sheard et al., 2009).

University students provide a particularly informative context for investigating this issue. Academic life requires sustained effort, concentration, emotional regulation, and long-term goal pursuit. At the same time, many students are regularly involved in physical activity or organized sport. Studying both general and sport-specific mental toughness in this population may clarify whether mental toughness reflects a broader dispositional tendency, a sport-specific adaptation, or both.

Mental toughness has often been conceptualized as partly trait-like and linked to stable individual differences in self-regulation and emotional adjustment (Clough et al., 2002; Gucciardi, 2014). Within the Big Five framework, conscientiousness is especially relevant because it includes discipline, organization, persistence, and goal-directed behavior, all of which overlap conceptually with core components of mental toughness. Recent empirical research has continued to support associations between mental toughness and broader personality characteristics, including Big Five traits, reinforcing the relevance of dispositional factors in sport-related mental toughness (Goddard et al., 2019). Neuroticism, by contrast, reflects emotional instability, worry, and vulnerability to stress, and is therefore expected to show an inverse association with mental toughness (Costa and McCrae, 1992; John et al., 2008).

This view is consistent with broader motivational models. Dweck's work on implicit theories suggests that individuals who view abilities as malleable are more likely to persist after setbacks and respond to difficulty in an adaptive way (Dweck and Leggett, 1988; Dweck, 2000, 2006). Similarly, grit has been defined as perseverance and passion for long-term goals and shares conceptual overlap with the commitment and persistence dimensions of mental toughness (Duckworth et al., 2007). These perspectives support the view that mental toughness should be examined in relation to broader dispositional and self-regulatory tendencies.

Sport participation may be relevant to mental toughness because regular engagement in training and exercise involves repeated exposure to effort, persistence, and behavioral regulation. These processes are conceptually aligned with mental toughness and may contribute to its development or expression over time. Previous research has shown that physical activity is associated with better psychological wellbeing, whereas sedentary behavior is associated with poorer adjustment and lower resilience-related outcomes (Belcher et al., 2021; Mah et al., 2011; Päivärinne et al., 2018). In the present study, training frequency and related behavioral indicators were examined as markers of sport participation and daily self-regulation.

In addition to sport participation, the present study also considered selected daily behavioral indicators, including mobile phone use and sedentary behavior. These variables were included as exploratory correlates because they may reflect broader patterns of self-regulation, lifestyle organization, and behavioral routines that are theoretically relevant to mental toughness. However, they were not central outcomes of the study and were therefore examined in an exploratory manner.

Mental toughness may also be relevant to academic functioning. Academic performance requires perseverance, planning, sustained attention, and the ability to tolerate setbacks over time. These characteristics overlap conceptually with both conscientiousness and mental toughness, suggesting that students with higher levels of mental toughness may also show better academic functioning (Clough and Strycharczyk, 2012; Dewhurst et al., 2019). In the present study, academic performance was considered as a secondary indicator of functioning in the university context.

Among students who practice sport regularly, competitive anxiety was examined as a secondary outcome relevant to sport-specific mental toughness. Competitive settings place athletes under evaluative pressure and may interfere with concentration, confidence, and emotional control. The Sport Competition Anxiety Test (SCAT) was developed to assess the tendency to experience anxiety in competitive sport situations (Martens, 1977). Recent meta-analytic evidence has confirmed the continuing relevance of competitive anxiety in sport psychology, showing that psychological interventions may effectively reduce anxiety in athletes (Ong and Chua, 2021). Because sport-specific mental toughness includes confidence, control, and effective functioning under pressure, higher levels of sport-specific mental toughness would be expected to be associated with lower competitive anxiety.

This expectation is consistent with previous literature linking mental toughness to more effective coping and emotional regulation in demanding sport environments (Gucciardi et al., 2008; Butt et al., 2010). This association was examined specifically within the subgroup of students engaged in regular sport practice. Previous research has established the relevance of mental toughness in both performance and adaptation, but fewer studies have examined general and sport-specific mental toughness within the same sample of university students.

To our knowledge, few studies have simultaneously examined general and sport-specific mental toughness in university students while integrating personality traits, behavioral indicators, and academic functioning. The present study examined general and sport-specific mental toughness in university students, focusing primarily on their associations with personality traits and sport participation variables. Academic performance was included as a secondary indicator of functioning in the university context, and competitive anxiety was examined among students engaged in regular sport practice.

Based on previous literature, the following hypotheses were formulated.

H1: General mental toughness and sport-specific mental toughness will be positively associated.

H2: Conscientiousness will be positively associated with both general and sport-specific mental toughness. Neuroticism will be examined as a potential negative correlate.

H3: Greater sport participation, particularly in terms of training frequency, will be associated with higher levels of general and sport-specific mental toughness. Academic performance will be examined as a secondary correlate of general mental toughness.

H4: Among students engaged in regular sport practice, sport-specific mental toughness will be negatively associated with competitive anxiety.

## 2 Materials and methods

### 2.1 Participants

The final analytic sample consisted of 239 university students. The sample included 86 men (36.0%), 147 women (61.5%), and 6 non-binary participants (2.5%), with a mean age of 22.00 years (SD = 3.54, range = 18-32 years). Participants were enrolled in different academic programs: Pedagogical Sciences (41.8%), Health Professions (25.1%), Legal Sciences (18.4%), and Sports Sciences (14.6%). Most participants were single (90.0%), whereas 5.9% were married and 4.2% were cohabiting. Participants were eligible if they were university students aged 18 years or older and provided informed consent to participate. Participants were excluded if they did not complete the questionnaire, did not provide informed consent, or showed insufficient effort responding during data screening.

Regarding sport participation, 155 students (64.9%) reported regular engagement in sport, whereas 84 (35.1%) did not. Among those practicing sport, 54.8% were involved in team sports, 19.4% in fitness-related activities, 12.9% in racket sports, and 12.9% in other sport disciplines. Additional descriptive information, including weekly training frequency, mobile phone use, and self-reported wellbeing indicators, is reported in Table 1.

**Table 1:** Demographic characteristics and lifestyle habits of participants (*N* = 239).

Variable	Category	*n*	%	*M*	SD
Gender	Male	86	36.0	—	—
	Female	147	61.5	—	—
	Non-binary	6	2.5	—	—
Age		—	—	22.00	3.54
Faculty	Pedagogical Sciences	100	41.8	—	—
	Health Professions	60	25.1	—	—
	Legal Sciences	44	18.4	—	—
	Sports Sciences	35	14.6	—	—
Marital status	Single	215	90.0	—	—
	Married	14	5.9	—	—
	Cohabiting	10	4.2	—	—
Sports practice	Yes	155	64.9	—	—
	No	84	35.1	—	—
Sport type	Team sports	85	54.8	—	—
	Fitness	30	19.4	—	—
	Racket sports	20	12.9	—	—
	Other	20	12.9	—	—
Daily habits	Weekly training days	—	—	3.00	1.72
	Daily mobile use (hrs)	—	—	5.00	2.39
Wellbeing (1–10)	Health status	—	—	8.00	1.67
	Energy level	—	—	7.00	1.92
	Sadness/depression	—	—	4.00	2.40
	Number of friends	—	—	2.10	1.20

An *a priori* power analysis was conducted using G^*^Power 3.1 for multiple linear regression analyses. Assuming a medium effect size (*f*^2^ = 0.15), an alpha level of.05, a desired statistical power of.80, and five predictors, the required sample size was estimated to range between approximately 92 and 100 participants. The final full sample (*N* = 239) therefore exceeded the minimum required size for the primary planned analyses. However, subgroup analyses involving only sport-practicing participants should be interpreted with appropriate caution due to the smaller subgroup sample size.

### 2.2 Procedure

Data were collected through an online survey administered via Google Forms. Participants were informed about the aims of the study, the voluntary nature of participation, the anonymity of responses, and their right to withdraw at any time. Informed consent was obtained before participation. The survey required approximately 5–10 minutes to complete.

The questionnaire included a demographic section followed by standardized psychological measures. The order of the questionnaires was randomized to reduce potential order effects. Data were collected anonymously.

The study was approved by the Ethics Committee of AOU Policlinico “G. Martino”: Approval Number: Prot. 0063899, January 2, 2025, Board Name: Comitato Etico Interaziendale Messina. The study was conducted according to the ethical principles of the Declaration of Helsinki and Good Clinical Practice principles were adopted.

The participants provided their written informed consent to participate in this study.

### 2.3 Measures

#### 2.3.1 General mental toughness

General mental toughness was assessed using the Mental Toughness Questionnaire-18 (MTQ-18) (Clough et al., 2002). The MTQ-18 is an 18-item self-report instrument rated on a 5-point Likert scale, designed to assess global mental toughness as a dispositional construct. Although derived from the broader 4C model (commitment, challenge, control, and confidence), the MTQ-18 is typically interpreted using a total score (Dagnall et al., 2019). In the present sample, internal consistency was good (Cronbach's α = 0.87).

#### 2.3.2 Sport-specific mental toughness

Sport-specific mental toughness was assessed using the Sport Mental Toughness Questionnaire (SMTQ) (Sheard et al., 2009). The SMTQ consists of 14 items rated on a 5-point Likert scale and provides an overall measure of mental toughness in sport contexts, with dimensions related to confidence, constancy, and control. In the present sample, internal consistency for the total score was good (Cronbach's α = 0.88). Although the SMTQ was originally developed for sport settings, it was administered to the full sample to allow comparison between general and sport-specific mental toughness across the university population. Nevertheless, because some participants were not regularly engaged in sport practice, additional sensitivity analyses were conducted within the subgroup of students reporting regular sport participation.

#### 2.3.3 Personality traits

Personality traits were assessed using the 10-item Big Five Inventory (BFI-10), originally developed by John et al. (1991, 2008). In the present study, the Italian adaptation of the BFI-10 validated by Guido et al. (2015) was used to assess extraversion, agreeableness, conscientiousness, neuroticism, and openness to experience. The BFI-10 includes two items for each trait and was developed as a brief measure of the Big Five personality dimensions. Following recommendations for very short scales, reliability was additionally considered in terms of inter-item associations and Spearman–Brown coefficients. Inter-item correlations ranged from *r* = 0.54 to *r* = 0.74, corresponding to Spearman–Brown coefficients ranging from.70 to.85.

#### 2.3.4 Competitive anxiety

Competitive trait anxiety was assessed using the Sport Competition Anxiety Test (SCAT) (Martens, 1977), a 15-item questionnaire measuring the tendency to perceive competitive situations as stressful or threatening. The SCAT was administered only to participants who reported regular sport practice. Internal consistency for the total score was acceptable (Cronbach's α = 0.70).

#### 2.3.5 Demographic, academic, and behavioral variables

Participants also completed a demographic questionnaire including age, gender, faculty, marital status, sport participation, sport type, weekly training frequency, daily mobile phone use, sedentary behavior, perceived health, energy level, sadness/depression, number of friends, and academic performance. Academic performance was assessed through participants' self-reported grade average across all university examinations completed at the time of participation. In the Italian university system, exam grades range from 18 to 30, with 30 cum laude coded as 31. Regular sport practice was assessed through a self-report item asking participants whether they engaged in sport activities on a regular basis (yes/no). These variables were included to characterize the sample and to examine additional associations between mental toughness, academic functioning, and behavioral indicators.

#### 2.3.6 Language of administration and scale adaptation

All questionnaires were administered in Italian. For personality traits, the validated Italian version of the BFI-10 was used (Guido et al., 2015). For the MTQ-18, SMTQ, and SCAT, no validated Italian versions were available to our knowledge; therefore, the instruments were translated into Italian and reviewed by bilingual researchers to ensure semantic clarity and conceptual equivalence with the original versions. Internal consistency was examined in the present sample for all scales. However, the factorial validity of the translated versions was not formally examined in the present study, and this issue is acknowledged as a limitation.

### 2.4 Data screening

Cases showing insufficient effort responding (IER) were identified using a multi-step screening procedure. Specifically, a long-string index was computed, defined as the maximum number of identical consecutive responses across the MTQ-18 and SMTQ items. Based on the length of the scales, a threshold of 10 identical consecutive responses was used; participants exceeding this threshold were flagged for manual review. In addition, intra-individual response variability (IRV) was calculated to identify invariant response patterns, with IRV values below 0.20 considered indicative of insufficient response variability. Based on these criteria, 13 cases (5.1% of the initial sample) were excluded due to non-differentiated response patterns, resulting in a final analytic sample of 239 participants.

### 2.5 Statistical analyses

All analyses were conducted using IBM SPSS Statistics (Version 25). Descriptive statistics (means, standard deviations, frequencies, and percentages) were computed for all variables. Sample characteristics are reported in Table 1, descriptive statistics in Table 2, correlation coefficients in Table 3, and regression models in Table 4.

**Table 2:** Descriptive statistics for the main study variables.

Variable	M	SD
General mental toughness (MTQ-18)	55.76	8.88
Sport-specific mental toughness (SMTQ)	43.08	7.57
Academic performance	24.16	3.46
Extraversion	6.97	2.38
Agreeableness	6.18	1.48
Conscientiousness	6.13	1.53
Neuroticism	6.40	1.18
Openness to experience	7.13	1.82
Competitive anxiety (SCAT)	28.44	7.89
Weekly training days	2.70	1.72
Daily mobile phone use	4.91	2.39
Sedentary time	6.48	3.77

Means and standard deviations are reported for the main study variables. SCAT scores refer to the subgroup of participants engaged in regular sport practice.

**Table 3:** Pearson correlations among the main study variables.

Variable	1	2	3	4	5	6	7	8	9	10	11	12
1. General mental toughness	—											
2. Sport-specific mental toughness	0.717^***^	—										
3. Academic performance	0.350^***^	0.030	—									
4. Extraversion	0.060	0.153^*^	0.031	—								
5. Agreeableness	0.242^***^	0.177^*^	−0.043	−0.030	—							
6. Conscientiousness	0.298^***^	0.354^***^	0.147^*^	0.090	−0.091	—						
7. Neuroticism	0.005	0.014	−0.141	−0.074	−0.093	0.085	—					
8. Openness to experience	0.059	0.155^*^	0.047	0.439^***^	−0.082	0.062	−0.081	—				
9. Competitive anxiety (SCAT)	−0.294^***^	−0.267^***^	−0.005	0.065	−0.057	−0.130^*^	0.130^*^	0.059	—			
10. Weekly training days	0.239^**^	0.307^***^	−0.082	0.051	−0.074	0.207	0.071	0.052	0.044	—		
11. Daily mobile phone use	−0.232^***^	−0.282^***^	−0.045	−0.025	−0.112	−0.211	−0.028	0.014	0.015	−0.050	—	
12. Sedentary time	−0.132	−0.147^*^	−0.039	0.043	−0.045	−0.154^*^	0.042	0.012	0.071	0.031	0.091	—

SCAT, Sport Competition Anxiety Test. Correlations involving SCAT were computed only among participants engaged in regular sport practice (*n* = 155). Correlations involving weekly training days were computed in the full sample, with non-sport participants coded as zero training days. *N* = 239 for all other correlations. ^*^p < 0.05. ^**^p < 0.01. ^***^p < 0.001.

**Table 4:** Multiple regression models predicting mental toughness and competetitive anxiety.

Outcome and predictor	*B*	SE	β	*t*	*p*	95% CI
Model 1: General mental toughness (MTQ−18)						
Extraversion	0.12	0.08	0.08	1.50	0.134	[−0.04, 0.28]
Agreeableness	0.28	0.09	0.21	3.11	0.002	[0.10, 0.46]
Conscientiousness	0.45	0.08	0.32	5.62	< 0.001	[0.29, 0.61]
Neuroticism	−0.08	0.10	−0.06	−0.80	0.424	[−0.28, 0.12]
Openness to experience	0.05	0.07	0.04	0.71	0.478	[−0.09, 0.19]
Model statistics			*F*_(5, 233)_ = 8.47	*p* < 0.001	*R^2^* = 0.16 Adj.	*R^2^* = 0.15
**Model 2: Sport–specific mental toughness (SMTQ)**						
Extraversion	0.09	0.07	0.08	1.28	0.201	[−0.05, 0.23]
Agreeableness	0.19	0.09	0.15	2.11	0.030	[0.01, 0.37]
Conscientiousness	0.52	0.07	0.38	7.43	< 0.001	[0.38, 0.66]
Neuroticism	−0.11	0.09	−0.09	−1.22	0.223	[−0.29, 0.07]
Openness to experience	0.03	0.08	0.02	0.37	0.711	[−0.13, 0.19]
Model statistics			*F*_(5, 233)_ = 10.36	*p* < 0.001	*R^2^* = 0.24, Adj	*R^2^* = 0.22
**Model 3: Competitive anxiety (SCAT)**						
Training days	−0.04	0.05	−0.05	−0.80	0.425	[−0.14, 0.06]
Sport experience	−0.02	0.06	−0.03	−0.33	0.741	[−0.14, 0.10]
Conscientiousness	−0.18	0.09	−0.14	−2.00	0.047	[−0.36, −0.004]
Neuroticism	0.33	0.10	0.25	3.30	0.002	[0.13, 0.53]
Sport–specific mental toughness	−0.41	0.09	−0.29	−4.55	< 0.001	[−0.59, −0.23]
Model statistics			*F* (_5, 149_) = 9.21	*p* < 0.001	*R^2^* = 0.22, Adj.	*R^2^* = 0.19

*N*, 239 for Models 1 and 2; *n*, 155 for Model 3; B, unstandardized coefficient; SE, standard error; β, standardized coefficient; CI, confidence interval. Predictors were entered simultaneously in all models. Assumptions of linearity, homoscedasticity, residual normality, independence of errors, and multicollinearity were checked and satisfied. All VIF values were below 2.1.

Pearson's correlation analyses were performed to examine associations among mental toughness, personality traits, sport participation-related variables, academic performance, and behavioral indicators. Multiple linear regression analyses were then conducted to identify personality predictors of general mental toughness (MTQ-18) and sport-specific mental toughness (SMTQ). In both models, the five Big Five personality traits, namely extraversion, agreeableness, conscientiousness, neuroticism, and openness to experience, were entered simultaneously as predictors. These models were conducted in the full sample. Age, gender, and faculty/academic program were not included as covariates in the main regression models, which were focused on personality predictors of mental toughness.

Sport participation, training frequency, academic performance, daily mobile phone use, and sedentary behavior were examined through correlational analyses. Academic performance was treated as a correlate of mental toughness rather than as a regression outcome. Daily mobile phone use and sedentary behavior were considered exploratory behavioral correlates. Because participants were enrolled in different academic programs, supplementary analyses examined the association between general mental toughness and academic performance while controlling for faculty/academic program.

To address potential concerns regarding the administration of the SMTQ to non-sport participants, supplementary sensitivity analyses were performed by repeating the main SMTQ-related correlation and regression analyses among participants engaged in regular sport practice only. Training frequency was treated as an ordinal self-report variable reflecting weekly training frequency. Non-sport participants were not coded as zero in subgroup analyses restricted to sport-practicing participants. Supplementary exploratory analyses were conducted to examine potential gender differences in mental toughness, sport participation variables, and competitive anxiety.

Among students engaged in regular sport practice, an additional multiple regression analysis was conducted with SCAT scores as the dependent variable. In this model, sport-specific mental toughness, conscientiousness, and neuroticism were entered as predictors, whereas training frequency and sport experience were treated as control variables. All predictors were entered simultaneously.

Assumptions of linearity, homoscedasticity, independence of errors, residual normality, and multicollinearity were checked separately for the full-sample regression models and for the sport-practicing subgroup model and were satisfied (VIF < 2.1). Normality of residuals was examined through Q–Q plots and the Shapiro–Wilk test. Statistical significance was set at *p* < 0.05.

## 3 Results

### 3.1 Descriptive statistics

Descriptive statistics for the main study variables are presented in Table 2. Mean scores were 55.76 (SD = 8.88) for general mental toughness and 43.08 (SD = 7.57) for sport-specific mental toughness. Means and standard deviations for personality traits, academic performance, competitive anxiety, and behavioral variables are also reported in Table 2.

### 3.2 Associations between general and sport-specific mental toughness

H1 was supported. General and sport-specific mental toughness were strongly positively associated, indicating substantial overlap between the two constructs while retaining domain-specific features, *r* = 0.717, *p* < 0.001 (see Table 3). Sensitivity analyses restricted to participants engaged in regular sport practice (*n* = 155) yielded comparable results. Specifically, the positive association between general mental toughness and sport-specific mental toughness remained strong and statistically significant, *r* = 0.729, *p* < 0.001, supporting the robustness of the findings within the sport-practicing subgroup.

### 3.3 Personality traits and mental toughness

H2 was partially supported. Conscientiousness was positively associated with both general mental toughness, *r* = 0.298, *p* < 0.001, and sport-specific mental toughness, *r* = 0.354, *p* < 0.001 (see Table 3). Neuroticism, examined as a potential negative correlate, was not significantly associated with either mental toughness measure. Sensitivity analyses restricted to students engaged in regular sport practice showed a comparable pattern of associations between personality traits and sport-specific mental toughness. In particular, conscientiousness remained positively associated with SMTQ scores, *r* = 0.372, *p* < 0.001.

### 3.4 Sport participation, training frequency, and academic performance

H3 received partial support. Training frequency was positively associated with both general mental toughness, *r* = 0.239, *p* < 0.001, and sport-specific mental toughness, *r* = 0.307, *p* < 0.001 (see Table 3). General mental toughness was also positively associated with academic performance, *r* = 0.350, *p* < 0.001. This association remained significant when controlling for faculty/academic program, partial *r* = 0.324, *p* < 0.001. Sensitivity analyses restricted to participants engaged in regular sport practice confirmed the positive association between training frequency and sport-specific mental toughness, *r* = 0.312, *p* < 0.001. Because these findings were based primarily on correlational analyses, they should be interpreted as associative rather than predictive relationships.

### 3.5 Competitive anxiety in students engaged in regular sport practice

H4 was supported. Among students engaged in regular sport practice, sport-specific mental toughness was negatively associated with competitive anxiety, *r* = −0.267, *p* < 0.001 (see Table 3).

### 3.6 Regression analyses

Regression results are presented in Table 4. The model predicting general mental toughness was significant, F _(5, 233)_ = 8.47, *p* < 0.001, explaining 16% of the variance (R^2^ = 0.16, adjusted R^2^ = 0.15). Conscientiousness emerged as the strongest positive predictor, β = 0.32, *p* < 0.001, followed by agreeableness, β = 0.21, *p* = 0.002.

The model predicting sport-specific mental toughness was also significant, F _(5, 233)_ = 10.36, *p* < 0.001, explaining 24% of the variance (*R*^2^ = 0.24, adjusted *R*^2^ = 0.22). Conscientiousness was the strongest positive predictor, β =.38, p < .001, whereas agreeableness showed a smaller positive contribution, β =.15, p =.030. Additional sensitivity analyses conducted within the subgroup of participants engaged in regular sport practice showed a substantially similar pattern of results. In particular, conscientiousness remained the strongest positive predictor of sport-specific mental toughness, β =.39, p < .001.

Among students engaged in regular sport practice, the model predicting competitive anxiety was significant, F(5, 149) = 9.21, p < .001, explaining 22% of the variance (R^2^ =.22, adjusted R^2^ =.19). Sport-specific mental toughness negatively predicted competitive anxiety, β = -.29, p < .001, whereas neuroticism positively predicted it, β =.25, p =.002. Conscientiousness also showed a small negative association, β = -.14, p =.047.

Additional analyses were conducted to examine whether sport-specific mental toughness explained variance in competitive anxiety beyond general mental toughness. When both MTQ-18 and SMTQ scores were entered simultaneously as predictors, sport-specific mental toughness remained a significant negative predictor of competitive anxiety, β = −0.28, *p* = 0.003, whereas general mental toughness was not a significant predictor, β = −0.04, *p* = 0.612. These findings suggest the presence of domain-specific variance associated with sport-related functioning.

### 3.7 Additional analyses

Additional exploratory analyses showed that daily mobile phone use was negatively associated with both general mental toughness, *r* = −0.232, *p* < 0.001, and sport-specific mental toughness, *r* = −0.282, *p* < 0.001. Sedentary behavior was negatively associated with sport-specific mental toughness, *r* = −0.147, *p* = 0.035 (see Table 3). Sensitivity analyses restricted to participants engaged in regular sport practice showed the same direction of associations for SMTQ-related exploratory findings. These variables were treated as secondary behavioral correlates, no correction for multiple testing was applied, and the findings should therefore be considered exploratory and interpreted cautiously. Exploratory analyses examining gender differences showed no substantial differences in general mental toughness, sport-specific mental toughness, or competitive anxiety across gender groups.

## 4 Discussion

The present study examined general and sport-specific mental toughness in university students, focusing primarily on their associations with personality traits and sport participation variables. Academic performance was considered as a secondary indicator of functioning in the university context, and competitive anxiety was examined among students engaged in regular sport practice. Overall, the findings showed that general and sport-specific mental toughness were strongly associated, that conscientiousness emerged as the most consistent personality correlate of both forms of mental toughness, and that training frequency was positively related to mental toughness. General mental toughness was also positively associated with academic performance, whereas sport-specific mental toughness was negatively associated with competitive anxiety in students engaged in regular sport practice.

### 4.1 Relationship between general and sport-specific mental toughness

A first relevant finding concerns the strong positive association between general and sport-specific mental toughness. The correlation between MTQ-18 and SMTQ corresponds to approximately 51% shared variance, indicating substantial overlap between the two measures while also leaving non-shared variance that may reflect domain-specific features. This result supports the view that the two constructs share common components, while still remaining conceptually distinguishable. This pattern is consistent with theoretical models that describe mental toughness as a multidimensional psychological resource that may operate across domains, but also take context-specific forms depending on the demands of the environment (Clough et al., 2002; Gucciardi et al., 2008; Sheard et al., 2009). Recent reviews and meta-analytic findings have similarly emphasized the multidimensional and context-dependent nature of mental toughness in sport and performance settings (Stamatis et al., 2020). In the present study, the strong correlation between MTQ-18 and SMTQ suggests that students who report higher levels of mental toughness in everyday life also tend to report greater confidence, constancy, and control in sport settings. At the same time, the non-shared variance suggests that the two instruments should not be treated as interchangeable.

### 4.2 Personality traits as correlates of mental toughness

A second major finding is that conscientiousness emerged as the clearest and most robust personality correlate of both general and sport-specific mental toughness. This result is theoretically coherent and aligns with previous literature linking mental toughness to persistence, discipline, commitment, and self-regulation (Clough et al., 2002; Fabio and Towey, 2018; Gucciardi, 2014). Conscientiousness includes precisely these characteristics, namely goal-directed behavior, organization, and sustained effort, and it is therefore not surprising that it showed both significant correlations and predictive effects in the regression models. This interpretation is also consistent with more recent empirical research linking mental toughness to broader dispositional personality characteristics, particularly conscientiousness and self-regulatory tendencies (Goddard et al., 2019). This finding strengthens the view that mental toughness is partly rooted in broader dispositional tendencies rather than being solely a sport-specific psychological resource. By contrast, neuroticism was not significantly associated with either general or sport-specific mental toughness in the main correlational analyses, although it did emerge as a relevant predictor of competitive anxiety. This pattern suggests that emotional instability may be more closely related to maladaptive emotional responses under competitive pressure than to lower levels of mental toughness *per se*.

### 4.3 Role of sport participation in mental toughness

The positive association between training frequency and both forms of mental toughness is another important result. Students who reported more frequent training also reported higher levels of both general and sport-specific mental toughness. This association is consistent with previous literature suggesting that regular engagement in sport may be linked to persistence, tolerance of discomfort, self-regulation, and behavioral discipline, all of which are conceptually aligned with mental toughness (Belcher et al., 2021; Päivärinne et al., 2018). However, given the cross-sectional design, the direction of this association cannot be determined. It is possible that regular training contributes to the development or expression of mental toughness, that students with higher mental toughness are more likely to maintain frequent training routines, or that both are influenced by other dispositional or contextual factors.

### 4.4 Mental toughness and academic functioning

The association between general mental toughness and academic performance is also noteworthy. Although academic functioning was treated as a secondary outcome in the present study, the finding that general mental toughness was positively associated with academic performance suggests that the relevance of mental toughness extends beyond sport settings. This result is theoretically plausible, as academic success requires sustained effort, frustration tolerance, planning, and long-term goal pursuit, all of which overlap conceptually with general mental toughness and conscientiousness (Clough and Strycharczyk, 2012; Dewhurst et al., 2019). In practical terms, this finding supports the idea that mental toughness may contribute to broader student adaptation and not only to sport-related functioning. At the same time, because academic performance was measured through self-report, this result should be interpreted with some caution and verified in future studies using more objective academic indicators.

### 4.5 Mental toughness and competitive anxiety

Among students engaged in regular sport practice, sport-specific mental toughness was negatively associated with competitive anxiety. This finding is consistent with previous work suggesting that mentally tougher athletes are better able to regulate pressure, maintain confidence, and function effectively in evaluative sport environments (Gucciardi et al., 2008; Butt et al., 2010). The regression results further reinforced this interpretation, showing that sport-specific mental toughness was negatively associated with competitive anxiety, whereas neuroticism was positively associated with it. This pattern is theoretically coherent and consistent with previous literature linking mental toughness to more adaptive responses under competitive pressure (Gucciardi et al., 2008; Butt et al., 2010). Recent meta-analytic evidence has continued to highlight the importance of competitive anxiety regulation in sport contexts and the relevance of psychological resources associated with effective coping under pressure (Ong and Chua, 2021). However, given the cross-sectional design, these findings should not be interpreted as evidence that mental toughness reduces competitive anxiety or functions as a protective factor in causal terms.

Taken together, the present findings support a view of mental toughness as a construct positioned at the intersection of personality and behavior. On the one hand, the strong contribution of conscientiousness suggests that mental toughness is partly grounded in relatively stable dispositional characteristics. On the other hand, the association with training frequency indicates that mental toughness is also linked to behavioral engagement in structured and effortful activities such as regular sport participation. This combination is theoretically important because it is consistent with the view that mental toughness is related to both dispositional tendencies and repeated engagement in demanding contexts.

These findings may also have practical implications for university and sport settings. If mental toughness is associated with both academic functioning and competitive anxiety, interventions targeting self-regulation, persistence, and emotional control may be worth examining across multiple domains of student life. In educational contexts, mental toughness-related skills may support academic adaptation and long-term goal pursuit. In sport contexts, they may help students cope more effectively with competition-related stress. At the same time, the central role of conscientiousness suggests that interventions should not assume that mental toughness develops independently of broader dispositional tendencies. Instead, future programs designed to address mental toughness may consider targeting concrete self-regulatory processes, such as planning, routine building, attentional control, and persistence under difficulty.

### 4.6 Exploratory behavioral findings

The additional associations involving daily mobile phone use and sedentary behavior should be interpreted cautiously because these variables were examined as exploratory correlates rather than primary study outcomes. Although the observed associations may tentatively suggest links between mental toughness and broader patterns of self-regulation and lifestyle organization, no causal conclusions can be drawn. Future studies should further investigate these behavioral correlates using more comprehensive and longitudinal designs.

## 5 Limitations

Several limitations should be acknowledged. First, the cross-sectional design does not allow causal inferences. It remains unclear whether sport participation contributes to the development of mental toughness, whether mentally tougher students are more likely to engage in regular sport participation, or whether both processes occur simultaneously.

Second, the study relied exclusively on self-report measures, which may have introduced common method variance and social desirability bias. This limitation is particularly relevant for the assessment of sport-specific mental toughness, as the SMTQ was administered within a mixed sample including students not regularly engaged in sport practice. Although supplementary analyses conducted within the sport-practicing subgroup yielded comparable findings, the interpretation of sport-specific mental toughness among non-sport participants should therefore be treated with caution.

Third, although all scales showed acceptable to good internal consistency in the present sample, the factorial validity of the Italian versions or adapted translations was not formally examined. Future studies should further evaluate the factorial validity and measurement invariance of the MTQ-18 and SMTQ in Italian university student samples.

Fourth, academic performance was assessed through self-reported grade average and may not fully correspond to official academic records. In addition, academic performance may not be fully comparable across academic programs, which may differ in grading practices and academic demands.

Fifth, although the final sample size was adequate for the primary planned analyses, findings involving subgroup analyses should still be interpreted with appropriate caution due to the smaller subgroup sample size.

Finally, although the study included several behavioral variables, sport participation was assessed using relatively simple indicators, primarily regular participation and weekly training frequency, and did not capture dimensions such as training intensity, duration, competitive level, or quality of training.

## 6 Future directions

Future research could extend the present findings in several ways. Longitudinal studies would help clarify the direction of the associations observed here, particularly those involving training frequency, academic functioning, and competitive anxiety. It would also be useful to examine whether interventions targeting self-regulation, growth-oriented beliefs, or performance routines can strengthen mental toughness in university populations. In addition, future work could incorporate more precise indicators of sport participation and objective academic outcomes. A further promising direction would be to explore whether the relationship between mental toughness and competitive anxiety differs across specific sport types, competitive levels, and stages of athletic development.

## 7 Conclusions

In conclusion, the present study shows that general and sport-specific mental toughness are closely related in university students and that both are associated with conscientiousness and regular sport participation. General mental toughness was also positively associated with academic performance, whereas sport-specific mental toughness was negatively associated with competitive anxiety among students engaged in regular sport practice. Overall, the findings suggest that mental toughness in university students is associated with both dispositional and behavioral factors.

## Data Availability

The raw data supporting the conclusions of this article will be made available by the authors, without undue reservation.
